# Congenital beta cell defects are not associated with markers of islet autoimmunity, even in the context of high genetic risk for type 1 diabetes

**DOI:** 10.1007/s00125-022-05697-3

**Published:** 2022-04-30

**Authors:** Rebecca C. Wyatt, William A. Hagopian, Bart O. Roep, Kashyap A. Patel, Brittany Resnick, Rebecca Dobbs, Michelle Hudson, EXE-T1D Consortium, Elisa De Franco, Sian Ellard, Sarah E. Flanagan, Andrew T. Hattersley, Richard A. Oram, Matthew B. Johnson

**Affiliations:** 1 Institute of Biomedical and Clinical Science, University of Exeter Medical School, Exeter, UK; 2 Pacific Northwest Research Institute, Seattle, WA USA; 3 Department of Internal Medicine, Leiden University Medical Center, Leiden, the Netherlands; 4 National Institute for Health Research Exeter Clinical Research Facility, Royal Devon and Exeter NHS Foundation Trust, Exeter, UK

**Keywords:** ER stress, HLA, Islet autoantibodies, Monogenic, Neonatal diabetes

## Abstract

**Aims/hypothesis:**

A key unanswered question in type 1 diabetes is whether beta cells initiate their own destruction or are victims of an aberrant immune response (beta cell suicide or homicide?). To investigate this, we assessed islet autoantibodies in individuals with congenital beta cell defects causing neonatal diabetes mellitus (NDM).

**Methods:**

We measured autoantibodies to GAD (GADA), islet antigen-2 (IA-2A) and zinc transporter 8 (ZnT8A) in 242 individuals with NDM (median age diagnosed 1.8 months [IQR 0.39–2.9 months]; median age collected 4.6 months [IQR 1.8–27.6 months]; median diabetes duration 2 months [IQR 0.6–23 months]), including 75 whose NDM resulted from severe beta cell endoplasmic reticulum (ER) stress. As a control cohort we also tested samples from 69 diabetes-free individuals (median age collected 9.9 months [IQR 9.0–48.6 months]) for autoantibodies.

**Results:**

We found low prevalence of islet autoantibodies in individuals with monogenic NDM; 13/242 (5.4% [95% CI 2.9, 9.0%]) had detectable GADA, IA-2A and/or ZnT8A. This was similar to the proportion in the control participants who did not have diabetes (1/69 positive [1.4%, 95% CI 0.03, 7.8%], *p*=0.3). Importantly, monogenic individuals with beta cell ER stress had a similar rate of GADA/IA-2A/ZnT8A positivity to non-ER stress aetiologies (2.7% [95% CI 0.3, 9.3%] vs 6.6% [95% CI 3.3, 11.5%] *p*=0.4). We observed no association between islet autoimmunity and genetic risk, age at testing (including 30 individuals >10 years at testing) or diabetes duration (*p*>0.4 for all).

**Conclusions/interpretation:**

Our data support the hypothesis that beta cell stress/dysfunction alone does not lead to the production of islet autoantibodies, even in the context of high-risk HLA types. This suggests that additional factors are required to trigger an autoimmune response towards beta cells.

**Graphical abstract:**

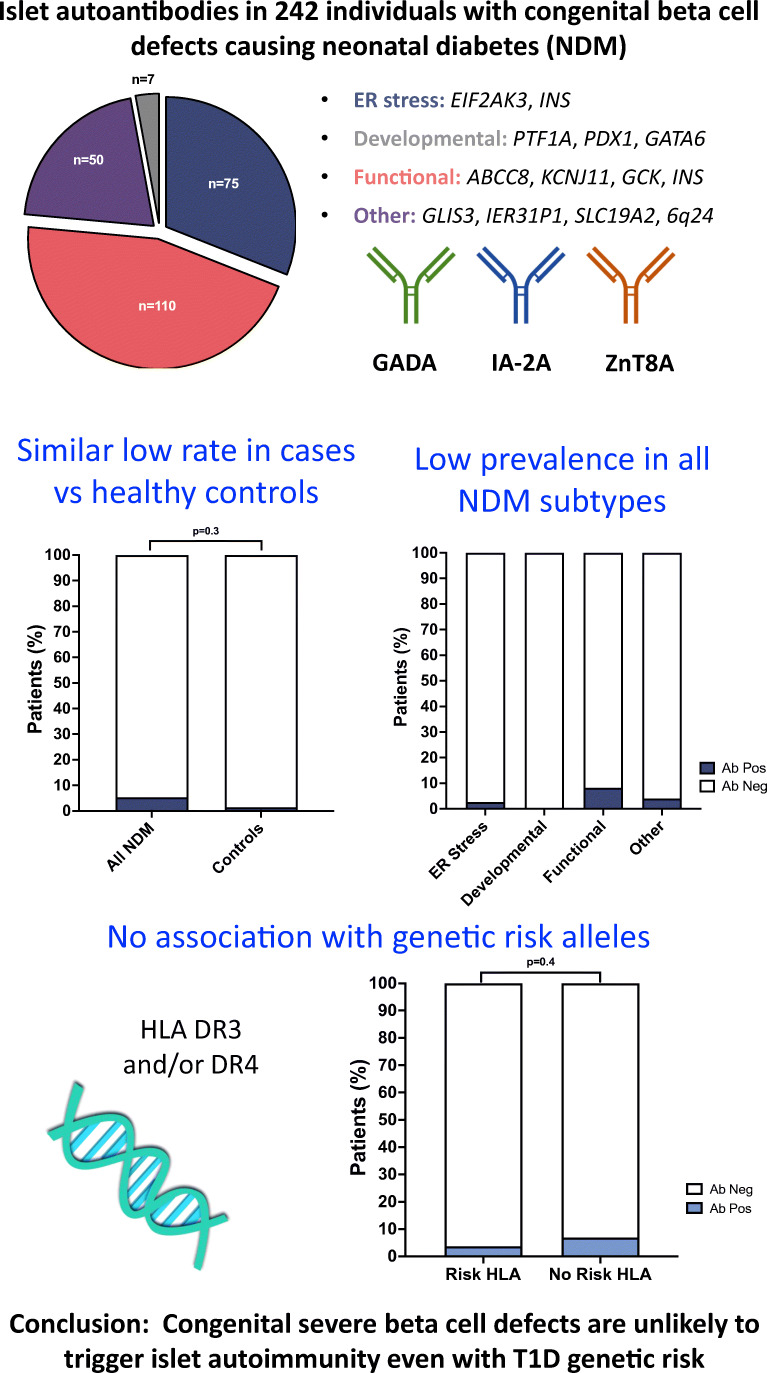

**Supplementary Information:**

The online version contains peer-reviewed but unedited supplementary material available at 10.1007/s00125-022-05697-3.

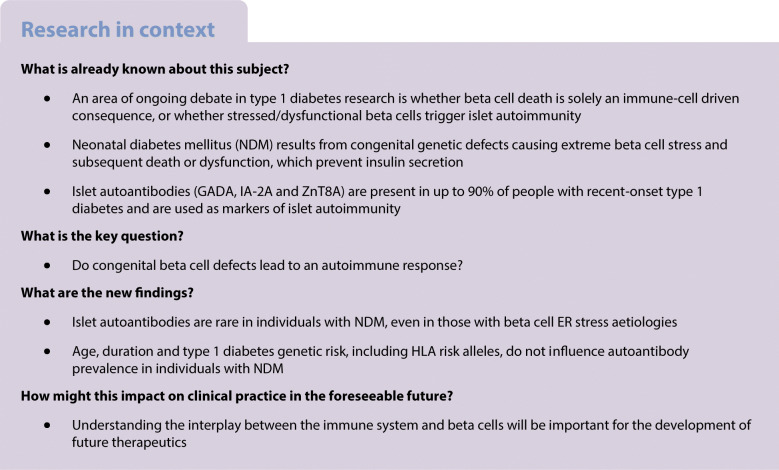



## Introduction

An ongoing debate in type 1 diabetes research is the question of beta cell homicide vs suicide; is beta cell death solely immune-cell driven, or do beta cells trigger their own demise [1, 2]? Several lines of evidence suggest a role for beta cell abnormalities in type 1 diabetes pathogenesis, including HLA class I hyperexpression [3] and irregular expression of immune genes under inflammatory conditions [4]. Reports of nonconventional, immunogenic polypeptides (e.g., defective ribosomal products [DRiPs]) acting as self-antigens in type 1 diabetes also suggests beta cells under endoplasmic reticulum (ER) stress may potentiate autoimmunity [5, 6].

Islet autoantibodies are present in up to 90% of individuals with recent-onset type 1 diabetes [7]. They are not considered pathogenic, but are markers of beta cell autoimmunity, evidenced by their use as markers of active autoimmunity in trials [8]. To examine whether congenital beta cell abnormalities are associated with islet autoantibodies, we measured autoantibodies to GAD (GADA), islet antigen-2 (IA-2A) and zinc transporter 8 (ZnT8A) in 242 individuals with monogenic neonatal diabetes mellitus (NDM). We assessed their association with NDM mechanistic subtype, HLA/polygenic risk for type 1 diabetes and age at sampling.

## Methods

We studied 242 individuals referred to our laboratory for genetic testing for NDM, in whom a pathogenic variant was identified and sufficient plasma was available for autoantibody testing (electronic supplementary material [ESM] Table 1: female participants: 136 [56%]; median age diagnosed 1.8 months [IQR 0.39–2.9 months]; median age collected 4.6 months [IQR 1.8–27.6 months]; median diabetes duration 2 months [IQR 0.6–23 months]) and 69 diabetes-free individuals (ESM Table 2: female participants: 38 [55%]; median age collected 9.9 months [IQR 9.0–48.6 months]). These samples came from unaffected relatives of individuals with monogenic diabetes who were sent for predictive testing and were found not to have inherited the pathogenic variant. They did not have a family history of type 1 diabetes and their risk of islet autoimmunity was therefore equivalent to the background population risk.

Autoantibodies to GAD, IA-2 and ZnT8 were measured by RIA at the Pacific Northwest Diabetes Research Institute, USA, who are participants of the Islet Autoantibody Standardisation Program. Positivity thresholds were defined by the 99th centile of index scores in 200 healthy control participants.

Individuals were categorised into mechanistic subtypes as follows: beta cell ER stress (biallelic *EIF2AK3* or heterozygous *INS* missense mutations [*n*=75], which have been assessed by mechanistic studies [9]); a functional defect (gain-of-function *ABCC8* or *KCNJ11*, biallelic *GCK* and biallelic *INS* variants [*n*=110]); pancreatic developmental defects (*PTF1A*, *PDX1* and *GATA6* variants [*n*=7]); or other (*GLIS3*, *IER3IP1* and *SLC19A2* variants or *6q24* methylation defect [*n*=50]) (see ESM Table 3 for detailed descriptions of genetic subtypes). We also grouped participants by sampling age: <4 months (*n*=106); 4 months–1 year (*n*=53); 1–5 years (*n*=28); 5–10 years (*n*=16); and ≥10 years (*n*=30) (ESM Fig. 1).

Where sufficient DNA was available (230/242) participants were also assessed for their type 1 diabetes genetic risk score (T1D-GRS) as described previously [10], and HLA-DR status.

We used the Mann–Whitney *U* test for continuous variables and Fisher’s exact test for categorical variables. Our study was powered to detect a 10% difference in antibody prevalence of the entire cohort compared with the control population and 30% difference in antibody prevalence between subcategories at 80% power and 5% significance.

All study participants gave informed consent or assent was obtained where children were too young and parental consent was provided, in accordance with the declaration of Helsinki. This study was approved by the Genetic Beta Cell Research Bank, Exeter, UK. Ethical approval was provided by the North Wales Research Ethics Committee, UK (IRAS project ID 231760).

## Results

**Fig. 1:**
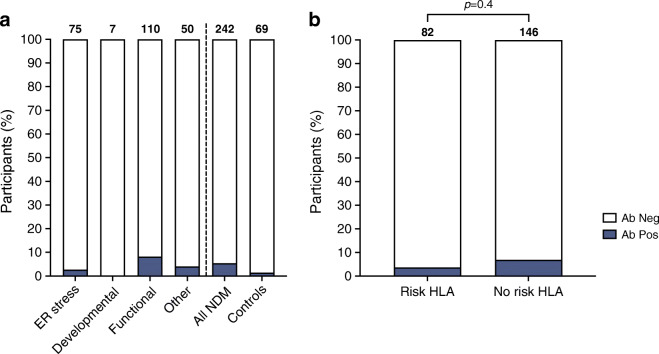
Proportion of individuals positive for islet autoantibodies in those with a congenital monogenic beta cell defect leading to ER stress (*n*=75), impacting beta cell development (*n*=7), function (*n*=110) or other (*n*=50) and in diabetes-free control participants (*n*=69) (**a**). Proportion of individuals with a congenital monogenic beta cell defect positive for islet autoantibodies in those with (*n*=82) or without (*n*=146) an HLA risk allele (**b**). Antibody positive individuals (*n*=13) were defined as exceeding the 99th centile of index scores in 200 healthy control participants for GADA, IA-2A and/or ZnT8A. Ab neg, antibody negative; Ab pos, antibody positive

We found low prevalence of islet autoantibodies in all mechanistic categories of NDM (Fig. 1a). Out of 242 participants, 13 (5.4% [95% CI 2.9, 9.0%]) had GADA (*n*=4 [1.7%]), IA-2A (*n*=6 [2.5%]) and/or ZnT8A (*n*=4 [1.7%]), compared with one individual who was positive for IA-2A out of 69 diabetes-free individuals (1.4% [95% CI 0.03, 7.8%] *p*=0.3).

To account for duration effects which could result in lower seropositivity over time, we also conducted a subanalysis of participants with <24 months duration. We found a similar prevalence of autoantibodies: 10/175 (5.7% [95% CI 2.8, 10.3%]) had GADA, IA-2A and/or ZnT8A.

We did not observe any associations between seropositivity and mechanistic subtype, T1D-GRS, age at diagnosis, birthweight or sex (*p*>0.4 for all comparisons). Individuals with beta cell ER stress had a similar rate of positivity to other aetiologies (2/75 [2.7%, 95% CI 0.3, 9.3%] vs 11/167 [6.6%, 95% CI 3.3, 11.5%] *p*=0.4).

Age at sampling also did not influence autoantibody positivity in this cohort. Of 106 very young patients (<4 months), 7 (6.6% [95% CI 2.7, 13.1%]) were positive for GADA, IA-2A and/or ZnT8A compared with 1/53 (1.9% [95% CI 0.04, 10.0%] *p*=0.3), 4/28 (14.3% [95% CI 4.0, 32.7%] *p*=0.2), 0/16 (*p*=0.6) and 1/30 (3.3% [95% CI 0.08, 17.2%] *p*=0.7) of those aged 4 months–1 year, 1–5 years, 5–10 years and ≥10 years, respectively (ESM Fig. 1b).

Positivity was not linked with HLA risk alleles; 3/82 (3.7% [95% CI 0.7, 10.3%]) participants with HLA-DR3 and/or DR4 had detectable islet autoantibodies compared with 10/146 (6.8% [95% CI 3.3, 12.2%]) without DR3 or DR4 (*p*=0.4). Of these, 3/3 and 7/10 individuals were aged <5 years at sampling (Fig. 1b). Of the 75 participants with a beta cell ER stress causing variant, 26 (34.7% [95% CI 24.0, 46.5%]) had an HLA risk allele, 1 (3.8% [95% CI 0.1, 19.6%]) of which was positive for IA-2A.

One participant (age at diagnosis 1.6 months, duration 1.3 months) was GADA and IA-2A positive, but was heterozygous for the known *KCNJ11* pathogenic variant, p.(Arg201His) [11]. They had low T1D-GRS (5th centile of type 1 diabetes control group), low birthweight (−2.2 SDs) and successfully transferred from insulin to sulfonylurea treatment after diagnosis.

## Discussion

We did not see a strong association between monogenic beta cell defects, including those causing beta cell death resulting from ER stress, and an islet-specific humoral response as measured against three islet autoantigens. Autoantibody prevalence was similar in our diabetes-free control participants and monogenic NDM cohorts. Our data do not negate evidence indicating that beta cell abnormalities, such as incorrect insulin processing [12], HLA class I hyperexpression [3] and irregular expression of immune genes [4] contribute to the pathogenesis of type 1 diabetes. Nevertheless, we show that severe beta cell stress/dysfunction, even in the context of high-risk HLA types, is unlikely to be sufficient to cause islet autoimmunity as measured by autoantibodies. This suggests that additional factors are necessary to initiate islet autoimmunity.

We defined autoimmunity by the presence of any of three accepted type 1 diabetes islet autoantibodies (GADA, IA-2A and ZnT8A). We cannot rule out novel autoantibodies specific to alternative autoantigens [5]. Indeed, defective ER function (e.g., caused by coding *INS* variants) can lead to accumulation of aberrantly processed molecules (such as insulin or DRiPs), which could function as neo-autoantigens. These specific misfolded *INS* proteins in individuals with missense variants could lead to specific autoantibodies which we are unable to detect. We are also unable to assess insulin autoimmunity in our cohort as all participants were insulin treated, meaning we would be unable to distinguish between insulin autoantibodies (IAA) recognising exogenous insulin (immunity) and IAA to endogenous insulin (autoimmunity) [13, 14].

Our study is cross-sectional; we cannot rule out the development of autoantibodies after participants were sampled. Islet autoantibodies often precede diagnosis with seroconversion peaking in early childhood (<5 years) [15]. The low prevalence in very young individuals may reflect the inefficiency of antibody production by the developing immune system. Although we do not have longitudinal samples, age at sampling ranged from 1 week to 58 years (ESM Table 1), and we saw no association between positivity and age at sampling.

Despite our best attempts at follow-up, we are also unable to exclude coincidental islet autoimmunity and type 1 diabetes in one individual with multiple autoantibodies. Their clinical presentation was in keeping with monogenic NDM and their diabetes remained controlled by sulfonylurea treatment 3 months after insulin was discontinued, supporting that they did not have severe insulin deficiency at the time of last follow-up (7 months), but it is possible that they have subsequently progressed to this.

To our knowledge, this is the largest assessment of evidence of autoimmunity in individuals with congenital beta cell defects causing NDM. A 2007 study of 11 individuals with NDM caused by pathogenic *KCNJ11* variants found that of nine individuals with long duration (>10 years)*,* 5 (56%) had at least one islet antibody [16]. In our cohort, 1 of 30 participants with ≥10 years duration (who had a pathogenic *INS* missense variant) was positive for an islet autoantibody (IA-2A). It is possible that this disparity in prevalence is attributable to the different autoantibodies tested in each study, positivity thresholds, testing methodologies used or the numbers of individuals assessed.

Our study focused on investigating the humoral autoimmune response in NDM. Islet autoantibodies are markers of autoimmunity rather than pathogenic, and future work could look for evidence of T cell mediated beta cell autoimmunity. Alternatively, it may be possible to identify novel autoantibodies to beta cell antigens which are specific to NDM, such as misfolded insulin epitopes found in individuals with monoallelic dominant *INS* variants.

We found 5.4% of participants with monogenic NDM were positive for at least one of either GADA, IA-2A or ZnT8A. Additionally, individuals with NDM due to monogenic autoimmunity commonly have islet autoantibodies [17]. The presence of islet autoantibodies should therefore not prevent comprehensive genetic testing for NDM in patients diagnosed aged <6 months.

In conclusion, we found low prevalence of islet autoantibodies in participants with a congenital beta cell defect, including those with severe beta cell ER stress. The number of these individuals was not significantly higher than seen in age and geographically matched diabetes-free control participants, implying that beta cell stress/dysfunction in isolation is unlikely to trigger beta cell autoimmunity.

## Supplementary information

ESM 1 (PDF 415 kb)

## Data Availability

Access to data is open only through collaboration. Requests for collaboration will be considered following an application to the Genetic Beta Cell Research Bank (https://www.diabetesgenes.org/current-research/genetic-beta-cell-research-bank/). Contact by email should be directed to the Lead Nurse, Bridget Knight (b.a.knight@exeter.ac.uk).

## Abbreviations

DRiPS: Defective ribosomal products
ER: Endoplasmic reticulum
GADA: GAD antibody
IA-2A: Islet antigen-2 antibody
IAA: Insulin autoantibodies
NDM: Neonatal diabetes mellitus
T1D-GRS: Type 1 diabetes genetic risk score
ZnT8A: Zinc transporter 8 antibody
