# Supplementary material for: Congenital beta cell defects are not associated with markers of islet autoimmunity, even in the context of high genetic risk for type 1 diabetes
Source: Diabetologia. 2022 Apr 30;65(7):1179–84. doi: 10.1007/s00125-022-05697-3 (PMC9174109; doi:10.1007/s00125-022-05697-3)
Supplement: Supplementary file 1 — (PDF 415 kb) [file 125_2022_5697_MOESM1_ESM.pdf]

Supplementary

EXE-T1D Consortium

Timothy J. McDonald[1,2], Timothy I.M. Tree[3,4], Clara Domingo-Vila[3], Suzanne Hammersley[1,5], Cate Speake[6], Michael N. Weedon[1]

1. Institute of Biomedical and Clinical Science, University of Exeter Medical School, UK
2. Blood Sciences, Royal Devon & Exeter NHS Foundation Trust, Exeter, U.K.
3. Department of Immunobiology, School of Immunobiology & Microbial Sciences, Kings College London, London, U.K.
4. NIHR Biomedical Research Centre Guys and St Thomas' NHS Foundation Trust and Kings College London, London, U.K.
5. National Institute for Health Exeter Research Clinical Research Facility, Royal Devon and Exeter NHS Foundation Trust, Exeter, U.K.
6. Diabetes Clinical Research Program, Benaroya Research Institute at Virginia Mason, Seattle, Washington.

ESM Table 1: Characteristics of the cohort of patients with congenital beta-cell defects. Mechanism groups defined as the underlying pathogenic mechanism of the genetic subtype. Variants are described against the canonical/longest transcript (*ABCC8*: NM_001287174.1, *KCNJ11*: NM_000525.3, *INS*: NM_000207.3, *SLC19A2*: NM_006996.3, *IER3IP1*: NM_016097.3, *GLIS3*: NM_001042413, *EIF2AK3*: NM_004836, *GCK*: NM_000162.5, *GATA6*: NM_005257.4, PTF1A: NM_178161.3, PDX1: NM_000209.3
T1D-GRS = type 1 diabetes genetic risk score, presented as centile of 1800 type 1 diabetes (T1D) controls from the Wellcome Trust Case Control Consortium [1]. Rows highlighted in green are autoantibody positive.

Patient	Mechanism	Gene	Pathogenic Variant	Islet autoantibody status	T1D-GRS Centile of T1D controls	Age at Sampling (months)	Duration (months)	Sex	Country of Origin
1	Developmental	*PTF1A*	p.?/p.? Chr10(Hg19):g.23508124-?_23508633+?del/g.23508124-?_23508633+?	Antibody negative	2	0.8	0.3	Female	UAE
2	Developmental	*PDX1*	p.(Arg173Pro)/p.(Arg173Pro)	Antibody negative	2	74.7	72.4	Female	India
3	Developmental	*PTF1A*	p.?/p.? Chr10(Hg19):g.23508437A>G/g.23508437A>G	Antibody negative	3	14.2	11.5	Female	Turkey
4	Developmental	*PTF1A*	p.?/p.? Chr10(Hg19):g.23508437A>G/g.23508437A>G	Antibody negative	16	1.1	1.1	Male	Turkey
5	Developmental	*PDX1*	p.(Ala152Gly)/p.(Ala152Gly)	Antibody negative	21	10.2	10.1	Male	India
6	Developmental	*PDX1*	p.(Lys163Arg)/p.(Lys163Arg)	Antibody negative	22	1.8	0.4	Male	India
7	Developmental	*GATA6*	p.(Ala302fs)/N c.899_902dupTGGC/N	Antibody negative	NA	0.6	0.2	Female	England
8	ER Stress	*INS*	p.(Arg89Cys)/N	Antibody negative	0.05	6.1	1.0	Female	Ukraine
9	ER Stress	*EIF2AK3*	p.(Ser991Asn)/p.(Ser991Asn)	Antibody negative	0.05	199.3	198.0	Male	Kuwait
10	ER Stress	*INS*	p.(Gly32Ser)/N	Antibody negative	0.05	0.0	0.0	Male	Venezuela

11	ER Stress	*INS*	p.(Gly75Cys)/N	Antibody negative	0.05	323.7	321.9	Male	England
12	ER Stress	*EIF2AK3*	p.(Leu863*)/p.(Leu863*)	Antibody negative	1	48.7	46.7	Female	Sri Lanka
13	ER Stress	*INS*	p.(Ala22Pro)/N	Antibody negative	1	5.2	2.4	Female	Kazakhstan
14	ER Stress	*INS*	p.(Arg89Cys)/N	Antibody negative	1	150.2	148.3	Male	Jordan
15	ER Stress	*INS*	p.(Phe48Cys)/N	Antibody negative	1	2.9	0.8	Male	Thailand
16	ER Stress	*EIF2AK3*	p.(Glu524*)/p.(Glu524*)	Antibody negative	1	1.8	1.1	Male	UAE
17	ER Stress	*INS*	p.(Gly32Ser)/N	Antibody negative	1	151.5	146.9	Female	England
18	ER Stress	*EIF2AK3*	p.(Cys531fs)/p.(Cys531fs) c.1591dup)/c.1591dup	Antibody negative	2	1.8	0.5	Male	India
19	ER Stress	*INS*	p.(Gly32Ser)/N	Antibody negative	2	2.4	0.6	Male	India
20	ER Stress	*EIF2AK3*	p.(Glu371*)/p.(Glu371*)	Antibody negative	2	100.7	97.2	Female	India
21	ER Stress	*EIF2AK3*	p.?/p.? c.1647+2T>A/c.1647+2T>A	Antibody negative	2	1.9	0.5	Male	Sudan
22	ER Stress	*INS*	p.(Gly32Ser)/N	Antibody negative	2	11.6	6.8	Female	Turkey
23	ER Stress	*INS*	p.(Ser101Cys)/N	Antibody negative	3	2.1	2.0	Male	Turkey
24	ER Stress	*EIF2AK3*	p.(Gln333*)/p.(Gln333*)	Antibody negative	3	8.2	7.2	Male	Turkey
25	ER Stress	*INS*	p.(Cys96Tyr)/N	Antibody negative	3	13.0	7.9	Female	India
26	ER Stress	*INS*	p.(Arg89Cys)/N	Antibody negative	3	181.0	175.0	Female	England
27	ER Stress	*INS*	p.(Cys96Tyr)/N	Antibody negative	3	52.4	48.5	Male	Ukraine
28	ER Stress	*INS*	p.(Tyr50Cys)/N	Antibody negative	3	0.0	0.0	Male	Austria
29	ER Stress	*EIF2AK3*	p.(Arg637*)/p.(Arg637*)	Antibody negative	4	81.6	80.1	Male	Sudan

30	ER Stress	*EIF2A K3*	p.(Asn999fs)/N c.2989_2990dup/N	Antibody negative	4	106.9	103.2	Female	Thailand
31	ER Stress	*EIF2A K3*	p.(Arg632Trp)/p.(Arg632Trp)	Antibody negative	4	4.5	1.1	Female	Morocco
32	ER Stress	*INS*	p.(Pro9Arg)/N	Antibody negative	4	2.0	0.9	Male	Vietnam
33	ER Stress	*EIF2A K3*	p.(Arg903*)/(p.Arg903*)	Antibody negative	4	2.3	0.5	Female	Kosovo
34	ER Stress	*EIF2A K3*	p.(Ile650Thr)/p.(IleI650Thr)	Antibody negative	5	3.9	2.5	Male	Israel
35	ER Stress	*EIF2A K3*	p.(Tyr989*)/p.(Tyr989*)	Antibody negative	5	0.0	0.0	Female	Sudan
36	ER Stress	*EIF2A K3*	p.(Glu371*)/p.(Glu371*)	Antibody negative	5	2.0	0.3	Male	India
37	ER Stress	*INS*	p.(Cys96Arg)/N	Antibody negative	5	7.2	2.6	Male	Argentina
38	ER Stress	*INS*	p.(Leu39Pro)/N	Antibody negative	5	0.8	0.6	Male	Germany
39	ER Stress	*INS*	p.(Arg89Cys)/N	Antibody negative	5	6.5	1.9	Female	Germany
40	ER Stress	*INS*	p.(Tyr50Cys)/N	Antibody negative	5	362.0	359.2	Male	Austria
41	ER Stress	*INS*	p.(Arg89Cys)/N	Antibody negative	6	3.3	0.3	Male	England
42	ER Stress	*INS*	p.(Cys96Arg)/N	Antibody negative	6	163.9	160.7	Female	India
43	ER Stress	*INS*	p.(Leu39His)/N	Antibody negative	7	73.4	73.3	Female	Saudi Arabia
44	ER Stress	*INS*	p.(Gly32Ser)/N	Antibody negative	7	6.0	1.0	Female	Vietnam
45	ER Stress	*INS*	p.(Lys105Pro)/N	Antibody negative	7	3.6	0.6	Male	England
46	ER Stress	*EIF2A K3*	p.(Gly1010Asp)/p.(Gly1010A sp)	Antibody negative	7	4.6	3.2	Male	Jordan
47	ER Stress	*INS*	p.(Gly32Ser)/N	Antibody negative	8	5.1	0.5	Female	Ukraine
48	ER Stress	*INS*	p.(Cys96Arg)/N	IA-2A positive	8	417.4	411.4	Female	Argentina

49	ER Stress	*EIF2A K3*	p.(Arg1064Gln)/(p.Arg1064G ln)	Antibody negative	10	5.7	1.1	Female	India
50	ER Stress	*EIF2A K3*	p.(Arg1064*)/p.(Arg1064*)	Antibody negative	10	3.6	2.0	Female	Lebanon
51	ER Stress	*INS*	p.(Lys35Pro)/N	Antibody negative	10	19.9	17.1	Male	Indonesia
52	ER Stress	*INS*	p.(Cys109Tyr)/N	Antibody negative	11	169.5	166.7	Female	Saudi Arabia
53	ER Stress	*INS*	p.(Cys109Tyr)/N	Antibody negative	11	169.5	166.7	Female	Saudi Arabia
54	ER Stress	*EIF2A K3*	p.(Arg826*)/p.(Arg826*)	Antibody negative	13	17.6	15.8	Female	Iran
55	ER Stress	*EIF2A K3*	p.(Thr905fs)/p.(Thr905fs) c.2713dup/c.2713dup	Antibody negative	13	2.4	0.6	Male	Egypt
56	ER Stress	*EIF2A K3*	p.(Asn420fs)/p.(Asn420fs) c.1259delA/c.1259delA	Antibody negative	15	8.1	6.2	Female	Saudi Arabia
57	ER Stress	*EIF2A K3*	p.(Cys531fs)/p.(Cys558fs) c.1591dupT/c.1670dupT	Antibody negative	16	0.0	0.0	Female	India
58	ER Stress	*INS*	p.(Arg89Cys)/N	Antibody negative	16	22.5	17.9	Male	Sri Lanka
59	ER Stress	*EIF2A K3*	p.(Arg1064*)/p.(Arg1064*)	Antibody negative	17	5.6	5.2	Male	India
60	ER Stress	*INS*	p.(Leu105Pro)/N	Antibody negative	21	6.9	2.3	Male	India
61	ER Stress	*EIF2A K3*	p.(Arg587Gln)/p.(Arg587Gln)	Antibody negative	21	2.4	1.3	Female	India
62	ER Stress	*INS*	p.(Arg89Cys)/N	Antibody negative	23	430.6	428.5	Female	UK
63	ER Stress	*EIF2A K3*	p.(Gln333*)/p.(Gln333*)	Antibody negative	24	14.2	12.8	Male	Turkey
64	ER Stress	*EIF2A K3*	p.(Gly956Glu)/p.(Gly956Glu)	Antibody negative	24	2.5	1.8	Female	India
65	ER Stress	*INS*	p.(Gly69Cys)/N	IA-2A positive	27	18.9	13.6	Male	Egypt
66	ER Stress	*EIF2A K3*	p.(Asp164fs)/p.(Glu421fs) c.492_495del/c.1254_1257del ins26	Antibody negative	28	3.7	0.8	Female	Ukraine

67	ER Stress	*INS*	p.(Cys95Arg)/N	Antibody negative	30	0.7	0.1	Female	England
68	ER Stress	*INS*	p.(Cys9Trp)/N	Antibody negative	31	3.4	0.1	Male	Turkey
69	ER Stress	*INS*	p.(Arg89Cys)/N	Antibody negative	33	44.8	42.7	Male	Sri Lanka
70	ER Stress	*EIF2AK3*	p.(Gly956Glu)/p.(Gly956Glu)	Antibody negative	35	116.5	114.6	Male	Turkey
71	ER Stress	*EIF2AK3*	p.(Gly1010Val)/p.(Gly1010Val)	Antibody negative	35	3.5	2.1	Male	Ukraine
72	ER Stress	*INS*	p.(Cys95Arg)/N	Antibody negative	37	333.1	327.1	Female	England
73	ER Stress	*INS*	p.(Cys96Tyr)/N	Antibody negative	38	102.9	99.2	Female	Iran
74	ER Stress	*EIF2AK3*	p.?/p.? c.1886_c.2818-?del/c.1886_c.2818-?del	Antibody negative	43	3.9	0.5	Female	Turkey
75	ER Stress	*EIF2AK3*	p.(Glu993Lys)/p.(Glu993Lys)	Antibody negative	44	0.0	0.0	Male	India
76	ER Stress	*EIF2AK3*	p.(Ile855fs)/p.(Ile855fs) c.2562dup/c.2562dup	Antibody negative	44	13.9	12.1	Female	Lebanon
77	ER Stress	*EIF2AK3*	p.?/p.? c.765-?_1162+?del/c.765-?_1162+?del	Antibody negative	NA	43.2	42.3	Female	Jordan
78	ER Stress	*EIF2AK3*	p.(Glu419fs)/p.(Gly1010Val) c.1254_1257delinsCGCAATCTGTCACTAACGCAATTGGT/c.3029G>T/c.3029G>T	Antibody negative	NA	6.0	3.2	Female	Ukraine
79	ER Stress	*EIF2AK3*	p.(Pro269fs)/p.(Pro269fs) c.802_803dup/c.802_803dup	Antibody negative	NA	3.0	1.0	Male	Sudan
80	ER Stress	*INS*	p.(Leu30Pro)/N	Antibody negative	NA	425.2	424.2	Female	England
81	ER Stress	*INS*	p.(Cys109Gly)/N	Antibody negative	NA	3.0	0.2	Male	Germany
82	ER Stress	*INS*	p.(Ser101Cys)/N	Antibody negative	NA	1.3	1.2	Male	India
83	Functional	*KCNJ11*	p.(Arg201His)/N	Antibody negative	0.05	1.6	0.0	Male	Austria

84	Functional	*KCNJ11*	p.(Leu17Pro)/N	Antibody negative	0.05	82.7	77.2	Male	Israel
85	Functional	*ABCC8*	p.(Pro1199Leu)/N	Antibody negative	0.05	1.8	0.4	Female	Turkey
86	Functional	*GCK*	p.(Ala188Thr)/p.(Ala188Thr)	Antibody negative	0.05	0.5	0.4	Male	Turkey
87	Functional	*KCNJ11*	p.(Lys170Arg)/N	Antibody negative	0.05	8.5	6.7	Female	Saudi Arabia
88	Functional	*KCNJ11*	p.(Arg201Cys)/N	Antibody negative	0.05	158.4	157.0	Male	USA
89	Functional	*KCNJ11*	p.(Glu227Lys)/N	Antibody negative	0.05	6.2	3.5	Male	Greece
90	Functional	*ABCC8*	p.(Arg1380His)/N	Antibody negative	0.05	60.1	59.4	Female	Malta
91	Functional	*KCNJ11*	p.(Val64Met)/N	Antibody negative	0.05	3.4	0.7	Female	USA
92	Functional	*KCNJ11*	p.(Arg201His)/N	ZnT8A positive	0.05	15.8	14.6	Male	Kazakhstan
93	Functional	*ABCC8*	p.(Asp212Glu)/N	Antibody negative	1	0.7	0.7	Female	Israel
94	Functional	*KCNJ11*	p.(Arg201His)/N	Antibody negative	1	358.4	355.9	Male	England
95	Functional	*GCK*	p.(Lys169Arg)/p.(Lys169Arg)	Antibody negative	1	1.1	1.1	Female	Turkey
96	Functional	*KCNJ11*	p.(Glu229Lys)/N	Antibody negative	1	14.1	8.8	Female	Ukraine
97	Functional	*KCNJ11*	p.(Arg201Cys)/N	Antibody negative	1	116.4	113.4	Female	Romania
98	Functional	*ABCC8*	p.(Tyr1176Cys)/N	Antibody negative	1	2.3	0.2	Female	Germany
99	Functional	*KCNJ11*	p.(Glu229Lys)/N	Antibody negative	1	55.2	52.4	Male	Romania
100	Functional	*KCNJ11*	p.(Arg201Cys)/N	Antibody negative	1	356.3	355.4	Male	Sweden
101	Functional	*KCNJ11*	p.(Val59Met)/N	Antibody negative	1	51.7	45.7	Male	England
102	Functional	*KCNJ11*	p.(Arg201His)/N	Antibody negative	1	343.5	340.8	Female	Thailand

103	Functional	*KCNJ11*	p.(Glu229Lys)/N	Antibody negative	1	3.7	1.4	Male	India
104	Functional	*ABCC8*	p.(Phe132Leu)/N	Antibody negative	1	3.5	0.7	Male	Morocco
105	Functional	*ABCC8*	p.(Phe132Leu)/N	Antibody negative	1	280.7	277.7	Female	England
106	Functional	*ABCC8*	p.(Leu225Pro)/N	Antibody negative	1	2.7	1.1	Male	USA
107	Functional	*ABCC8*	p.(Pro1199Ser)/N	Antibody negative	1	3.9	1.1	Female	India
108	Functional	*KCNJ11*	p.(Arg201Leu)/N	Antibody negative	1	4.8	1.1	Male	Lebanon
109	Functional	*KCNJ11*	p.(Arg50Pro)/N	IA-2A positive	2	2.8	0.8	Female	Sri Lanka
110	Functional	*KCNJ11*	p.(Val252Leu)/N	Antibody negative	2	44.2	42.9	Female	Iran
111	Functional	*ABCC8*	p.(Ala1264Val)/p.(Ala1264Val)	Antibody negative	2	3.6	1.0	Male	India
112	Functional	*ABCC8*	p.(Arg1380Cys)/N	Antibody negative	2	3.1	0.5	Male	India
113	Functional	*GCK*	p.(Ala449Thr)/p.(Ala449Thr)	Antibody negative	2	2.7	2.0	Male	Jordan
114	Functional	*KCNJ11*	p.(Arg201Cys)/N	Antibody negative	2	62.9	60.1	Female	Ukraine
115	Functional	*KCNJ11*	p.(His46Tyr)/N	Antibody negative	2	4.4	2.4	Female	Venezuela
116	Functional	*GCK*	p.(Arg43Cys)/p.(Arg43Cys)	Antibody negative	2	7.8	2.5	Male	India
117	Functional	*KCNJ11*	p.(Glu227Lys)/N	Antibody negative	3	8.8	8.4	Female	Jordan
118	Functional	*KCNJ11*	p.(Val59Met)/N	Antibody negative	3	3.3	3.2	Female	Romania
119	Functional	*KCNJ11*	p.(Arg201Cys)/N	Antibody negative	3	98.1	95.5	Female	England
120	Functional	*KCNJ11*	p.(Gly53Ser)/N	ZnT8A positive	3	35.5	32.0	Female	Egypt
121	Functional	*KCNJ11*	p.(Leu233Phe)/N	Antibody negative	3	234.4	230.7	Female	England

122	Functional	*KCNJ11*	p.(Val59Met)/N	Antibody negative	3	199.2	197.1	Female	England
123	Functional	*KCNJ11*	p.(Gly334Val)/N	Antibody negative	3	260.5	259.8	Female	England
124	Functional	*GCK*	p.(Cys382Tyr)/p.(Cys382Tyr)	ZnT8A positive	3	53.0	52.6	Male	Iran
125	Functional	*GCK*	p.(Ala449Thr)/p.(Ala449Thr)	Antibody negative	3	26.9	22.3	Male	Jordan
126	Functional	*KCNJ11*	p.(Arg201His)/N	Antibody negative	4	8.6	4.9	Female	Germany
127	Functional	*KCNJ11*	p.(Arg50Gln)/N	Antibody negative	4	6.2	0.3	Female	Sweden
128	Functional	*ABCC8*	p.(Ala110Thr)/N	Antibody negative	4	4.0	0.3	Female	Germany
129	Functional	*KCNJ11*	p.(Arg50Gln)/N	Antibody negative	5	7.7	5.4	Male	Vietnam
130	Functional	*KCNJ11*	p.(Arg201His)/N	GADA positive IA-2A positive	5	2.9	1.3	Female	India
131	Functional	*KCNJ11*	p.(Lys170Arg)/N	Antibody negative	6	5.0	2.5	Male	Peru
132	Functional	*KCNJ11*	p.(Gln52Arg)/N	Antibody negative	6	6.0	0.9	Female	India
133	Functional	*GCK*	p.(Ala188Thr/p.(Ala188Thr)	IA-2A positive	6	0.5	0.1	Female	England
134	Functional	*GCK*	p.(Ala449Thr/p.(Ala449Thr)	Antibody negative	6	27.6	23.0	Male	Jordan
135	Functional	*KCNJ11*	p.(Arg201His)/N	Antibody negative	6	483.8	481.9	Female	England
136	Functional	*KCNJ11*	p.(Glu227Lys)/N	Antibody negative	7	11.9	9.2	Male	Jordan
137	Functional	*ABCC8*	p.(Pro254Ser)/p.(Pro254Ser)	Antibody negative	7	6.7	5.6	Female	India
138	Functional	*ABCC8*	p.(Arg826Trp)/N	Antibody negative	7	2.2	0.4	Male	Sudan
139	Functional	*KCNJ11*	p.(Trp68Leu)/N	Antibody negative	8	1.7	0.1	Female	India

140	Functional	*ABCC8*	p.(Arg1380Cys)/N	Antibody negative	8	0.5	0.5	Female	South Africa
141	Functional	*KCNJ11*	p.(Glu322Lys)/N	Antibody negative	8	2.8	1.0	Male	Turkey
142	Functional	*ABCC8*	p.(Arg999Pro)/N	GADA positive	9	1.7	0.4	Male	Kenya
143	Functional	*KCNJ11*	p.(Lys185Thr)/N	Antibody negative	9	2.3	0.5	Female	Turkey
144	Functional	*KCNJ11*	p.(Glu179Lys)/N	Antibody negative	9	1.9	0.3	Female	Germany
145	Functional	*KCNJ11*	p.(Glu322Lys)/N	Antibody negative	9	4.1	2.3	Female	Venezuela
146	Functional	*KCNJ11*	p.(Arg201Cys)/N	Antibody negative	9	6.7	1.2	Female	UAE
147	Functional	*KCNJ11*	p.(Arg201His)/N	GADA positive	10	4.4	1.6	Male	Vietnam
148	Functional	*ABCC8*	p.(Asn262Lys)/p.(Asn262Lys)	Antibody negative	10	3.1	0.3	Female	Egypt
149	Functional	*KCNJ11*	p.(Gly53Asp)/N	Antibody negative	10	9.4	5.8	Female	Vietnam
150	Functional	*KCNJ11*	p.(Val59Met)/N	Antibody negative	10	71.8	70.9	Female	Ukraine
151	Functional	*GCK*	p.(Glu256fs)/p.(Glu256fs) c.764_767dup/c.764_767dup	Antibody negative	11	8.3	7.6	Female	India
152	Functional	*KCNJ11*	p.(Glu229Lys)/N	Antibody negative	11	37.9	36.0	Female	Kazakhstan
153	Functional	*KCNJ11*	p.(Arg201His)/N	Antibody negative	11	1.3	1.3	Male	Pakistan
154	Functional	*KCNJ11*	p.(Val59Met)/N	Antibody negative	11	5.7	2.5	Female	Turkey
155	Functional	*KCNJ11*	p.(His46Tyr)/N	Antibody negative	11	0.6	0.5	Female	Ukraine
156	Functional	*KCNJ11*	p.(Val59Met)/N	Antibody negative	12	1.6	0.7	Female	Turkey
157	Functional	*KCNJ11*	p.(Val59Met)/N	Antibody negative	12	40.5	39.2	Male	Jordan
158	Functional	*KCNJ11*	p.(Arg201Cys)/N	Antibody negative	13	3.3	0.3	Female	Ukraine

159	Functional	*ABCC8*	p.(Leu1169delinsValAlaLeuPhe)/N c.3505_3507delinsGTGGCCCTCTTT/N	Antibody negative	13	1.1	0.8	Female	Egypt
160	Functional	*KCNJ11*	p.(Arg201His)/N	Antibody negative	15	6.0	0.7	Female	India
161	Functional	*KCNJ11*	p.(Arg201Cys)/N	Antibody negative	15	191.2	188.5	Male	Belgium
162	Functional	*KCNJ11*	p.(Glu227Lys)/N	Antibody negative	15	63.2	60.9	Male	Jordan
163	Functional	*KCNJ11*	p.(Arg201His)/N	Antibody negative	17	10.1	6.4	Male	Bosnia and Herzegovina
164	Functional	*ABCC8*	p.(Asp209Glu)/N	Antibody negative	17	4.2	3.3	Female	Turkey
165	Functional	*ABCC8*	p.(His125Gln)/p.(Arg1183Trp)	Antibody negative	20	1.5	0.4	Male	Germany
166	Functional	*KCNJ11*	p.(Gln52Arg)/N	Antibody negative	25	14.3	14.2	Male	Romania
167	Functional	*GCK*	p.(Gly285Val)/p.(Gly285Val)	Antibody negative	25	3.4	0.4	Female	India
168	Functional	*KCNJ11*	p.(Glu227Lys)/N	Antibody negative	25	82.6	77.5	Male	Jordan
169	Functional	*KCNJ11*	p.(Trp68Gly)/N	Antibody negative	29	0.3	0.1	Female	Ireland
170	Functional	*KCNJ11*	p.(Arg201Cys)/N	Antibody negative	30	2.1	0.5	Female	Turkey
171	Functional	*ABCC8*	p.(Phe1164Leu)/p.(Phe1164Leu)	Antibody negative	32	4.9	2.2	Female	India
172	Functional	*KCNJ11*	p.(Glu322Lys)/N	Antibody negative	33	0.0	0.0	Female	Saudi Arabia
173	Functional	*KCNJ11*	p.(Gly53Ser)/N	Antibody negative	37	4.6	2.8	Male	Vietnam
174	Functional	*KCNJ11*	p.(Arg201His)/N	GADA positive	38	1.6	1.6	Female	Ukraine
175	Functional	*GCK*	p.(Ser375fs)/p.(Ser375fs) c.1121dup/c.1121dup	Antibody negative	40	4.9	0.3	Male	Pakistan
176	Functional	*KCNJ11*	p.(Glu227Lys)/N	Antibody negative	56	5.0	0.7	Female	Guatemala

177	Functional	*KCNJ11*	p.(Glu322Lys)/N	Antibody negative	60	27.6	23.0	Female	Venezuela
178	Functional	*KCNJ11*	p.(Val59Met)/N	Antibody negative	70	0.0	0.0	Female	Guatemala
179	Functional	*ABCC8*	p.(Arg1554Gln)/N	Antibody negative	71	1.4	1.1	Female	Austria
180	Functional	*ABCC8*	p.(Leu1426Pro)/p.(Leu1426Pro)	Antibody negative	72	0.0	0.0	Female	Honduras
181	Functional	*ABCC8*	p.(Gln211Lys)/N	Antibody negative	NA	9.2	7.8	Male	Kenya
182	Functional	*KCNJ11*	p.(Val59Met)/N	Antibody negative	NA	79.2	79.0	Male	England
183	Functional	*KCNJ11*	p.(Arg201His)/N	Antibody negative	NA	3.2	1.3	Female	Egypt
184	Functional	*KCNJ11*	p.(Arg201Cys)/N	Antibody negative	NA	4.4	0.7	Male	Ukraine
185	Other	*6q24*	Methylation defect	Antibody negative	0.05	0.7	0.3	Male	Turkey
186	Other	*6q24*	Methylation defect	Antibody negative	0.05	0.5	0.4	Female	UK
187	Other	*6q24*	Methylation defect	Antibody negative	0.05	0.6	0.2	Male	England
188	Other	*6q24*	Methylation defect	Antibody negative	0.05	0.6	0.6	Female	Turkey
189	Other	*6q24*	Methylation defect	Antibody negative	0.05	456.7	456.7	Female	Netherlands
190	Other	*6q24*	Methylation defect	IA-2A positive	1	1.0	0.8	Male	India
191	Other	*6q24*	Methylation defect	Antibody negative	1	382.3	380.9	Female	Belgium
192	Other	*6q24*	Methylation defect	Antibody negative	1	0.6	0.3	Female	Sweden
193	Other	*6q24*	Methylation defect	Antibody negative	1	0.9	0.9	Female	Ireland
194	Other	*6q24*	Methylation defect	ZnT8A positive	1	0.3	0.2	Female	Venezuela
195	Other	*6q24*	Methylation defect	Antibody negative	1	8.9	8.8	Female	Iran

196	Other	*6q24*	Methylation defect	Antibody negative	1	0.7	0.1	Female	Sweden
197	Other	*6q24*	Methylation defect	Antibody negative	1	1.6	0.5	Male	India
198	Other	*6q24*	Methylation defect	Antibody negative	1	1.3	1.2	Female	Germany
199	Other	*6q24*	Methylation defect	Antibody negative	1	0.3	0.2	Female	Germany
200	Other	*6q24*	Methylation defect	Antibody negative	1	0.8	0.6	Male	Germany
201	Other	*6q24*	Methylation defect	Antibody negative	1	1.3	1.3	Female	Sri Lanka
202	Other	*6q24*	Methylation defect	Antibody negative	2	0.9	0.7	Female	India
203	Other	*6q24*	Methylation defect	Antibody negative	2	292.5	292.1	Male	Hungary
204	Other	*6q24*	Methylation defect	Antibody negative	2	0.7	0.6	Male	Turkey
205	Other	*6q24*	Methylation defect	Antibody negative	3	0.9	0.8	Male	Belgium
206	Other	*6q24*	Methylation defect	Antibody negative	4	1.4	0.8	Male	Turkey
207	Other	*6q24*	Methylation defect	Antibody negative	4	0.3	0.3	Female	USA
208	Other	*6q24*	Methylation defect	Antibody negative	5	2.4	2.3	Female	Argentina
209	Other	*6q24*	Methylation defect	Antibody negative	5	1.6	0.7	Female	Vietnam
210	Other	*6q24*	Methylation defect	Antibody negative	6	1.3	0.5	Male	Vietnam
211	Other	*6q24*	Methylation defect	Antibody negative	7	12.9	12.9	Female	Turkey
212	Other	*6q24*	Methylation defect	Antibody negative	12	16.1	14.8	Male	India
213	Other	*6q24*	Methylation defect	Antibody negative	14	1.0	0.1	Female	Puerto Rico
214	Other	*6q24*	Methylation defect	Antibody negative	15	1.4	1.3	Male	Ukraine

215	Other	*6q24*	Methylation defect	Antibody negative	15	0.6	0.5	Female	Germany
216	Other	*6q24*	Methylation defect	Antibody negative	17	5.4	4.4	Male	Philippines
217	Other	*6q24*	Methylation defect	Antibody negative	17	0.0	0.0	Female	Canada
218	Other	*6q24*	Methylation defect	Antibody negative	18	1.2	0.5	Male	Vietnam
219	Other	*6q24*	Methylation defect	Antibody negative	23	1.3	1.2	Male	Turkey
220	Other	*6q24*	Methylation defect	Antibody negative	24	0.3	0.3	Female	England
221	Other	*6q24*	Methylation defect	Antibody negative	27	3.2	3.0	Female	India
222	Other	*6q24*	Methylation defect	Antibody negative	27	0.4	0.3	Female	England
223	Other	*6q24*	Methylation defect	Antibody negative	30	1.7	1.5	Male	Argentina
224	Other	*6q24*	Methylation defect	Antibody negative	37	0.3	0.1	Male	England
225	Other	*6q24*	Methylation defect	Antibody negative	38	1.0	0.5	Female	Morocco
226	Other	*6q24*	Methylation defect	Antibody negative	41	8.1	8.0	Male	USA
227	Other	*6q24*	Methylation defect	Antibody negative	42	0.5	0.4	Female	Sweden
228	Other	*6q24*	Methylation defect	Antibody negative	50	34.5	33.4	Female	Ukraine
229	Other	*6q24*	Methylation defect	Antibody negative	81	0.6	0.5	Female	Hong Kong
230	Other	*6q24*	Methylation defect	Antibody negative	NA	0.8	0.7	Male	Saudi Arabia
231	Functional	*INS*	p.?/N c.188-31G>A/N	Antibody negative	0	217.4	213.7	Female	USA
232	Other	*SLC19A2*	p.(Glu254*)/p.(Glu254*)	Antibody negative	1	9.3	4.2	Female	Honduras
233	Functional	*INS*	p.?/N c.188-31G>A/N	Antibody negative	1	289.2	288.5	Male	Venezuela

234	Functional	*INS*	p.?/N c.188-31G>A)/N	Antibody negative	2	3.3	1.0	Male	Portugal
235	Other	*GLIS3*	p.(Gly311fs)/p.(Gly311fs) c.932del/c.932del	Antibody negative	3	0.2	0.2	Female	England
236	Functional	*INS*	p.?/p.? c.-331C>G)/c.-331C>G	Antibody negative	4	5.1	4.8	Male	Saudi Arabia
237	Functional	*INS*	p.?/p.? c.-331C>G/c.-331C>G	Antibody negative	4	2.7	1.5	Female	Saudi Arabia
238	Functional	*INS*	p.?/N c.188-31G>A/N	Antibody negative	6	448.0	446.1	Male	Egypt
239	Other	*IER3IP 1*	p.(Leu78Pro)/p.(Leu78Pro)	Antibody negative	9	2.6	0.8	Female	Egypt
240	Functional	*INS*	p.(1_110del)/p.(1_110del) c.?_1_333+1_?del/c.?_-1_333+1_?del	Antibody negative	11	2.8	2.7	Male	Turkey
241	Functional	*INS*	p.?/N c.188-31G>A/N	Antibody negative	13	705.4	699.9	Female	Canada
242	Other	*SLC19 A2*	p.(Met401fs)/p.(Met401fs) c.1201_1202del/c.1201_1202 del	Antibody negative	71	5.3	0.7	Female	Guatemala

ESM Table 2: Characteristics of diabetes-free controls. Rows highlighted in green are autoantibody positive.

Patient	Autoantibody Status	Sex	Age at Sampling (months)	Country of origin
1	Antibody negative	Male	68.9	Turkey
2	Antibody negative	Female	16.5	England
3	Antibody negative	Female	45.9	Turkey
4	Antibody negative	Male	64.6	Turkey
5	Antibody negative	Male	8.0	Sri Lanka
6	Antibody negative	Female	61.4	Italy
7	Antibody negative	Male	40.1	Argentina
8	Antibody negative	Male	18.1	England
9	Antibody negative	Male	41.0	Saudi Arabia
10	Antibody negative	Female	33.6	India
11	Antibody negative	Male	2.8	England
12	Antibody negative	Female	48.3	Malta
13	Antibody negative	Male	0.2	Bulgaria
14	Antibody negative	Male	0.1	England
15	Antibody negative	Female	50.7	Sri Lanka
16	Antibody negative	Female	6.5	Serbia
17	Antibody negative	Female	64.9	Turkey
18	Antibody negative	Female	55.6	India
19	Antibody negative	Female	0.0	Canada
20	Antibody negative	Female	50.2	England
21	Antibody negative	Male	7.0	Sudan
22	Antibody negative	Female	57.2	Sri Lanka
23	Antibody negative	Female	68.2	England
24	Antibody negative	Female	44.3	Scotland
25	Antibody negative	Female	16.3	Bulgaria
26	IA-2A positive	Female	49.4	England
27	Antibody negative	Female	62.6	England
28	Antibody negative	Male	0.2	England
29	Antibody negative	Female	70.3	Saudi Arabia
30	Antibody negative	Male	52.4	Ireland
31	Antibody negative	Male	50.6	Turkey
32	Antibody negative	Female	61.8	England
33	Antibody negative	Female	23.2	Bulgaria
34	Antibody negative	Female	0.2	Austria
35	Antibody negative	Male	1.8	Oman
36	Antibody negative	Male	Unknown	Turkey
37	Antibody negative	Male	31.9	Italy
38	Antibody negative	Male	62.8	Netherlands
39	Antibody negative	Female	44.9	USA
40	Antibody negative	Male	34.8	England
41	Antibody negative	Male	0.1	England
42	Antibody negative	Female	52.7	England
43	Antibody negative	Female	29.5	England
44	Antibody negative	Female	9.2	USA
45	Antibody negative	Female	9.7	USA
46	Antibody negative	Male	9.6	USA
47	Antibody negative	Male	9.3	USA
48	Antibody negative	Male	9.0	USA
49	Antibody negative	Male	9.9	USA
50	Antibody negative	Female	9.7	USA
51	Antibody negative	Male	10.4	USA
52	Antibody negative	Female	9.9	USA

53	Antibody negative	Female	9.4	USA
54	Antibody negative	Female	9.0	USA
55	Antibody negative	Female	9.1	USA
56	Antibody negative	Male	9.5	USA
57	Antibody negative	Male	9.1	USA
58	Antibody negative	Female	9.5	USA
59	Antibody negative	Female	9.6	USA
60	Antibody negative	Female	9.8	USA
61	Antibody negative	Male	8.5	USA
62	Antibody negative	Male	9.1	USA
63	Antibody negative	Male	9.0	USA
64	Antibody negative	Female	9.1	USA
65	Antibody negative	Female	9.6	USA
66	Antibody negative	Male	8.7	USA
67	Antibody negative	Male	9.3	USA
68	Antibody negative	Female	9.4	USA
69	Antibody negative	Female	9.8	USA

ESM Table 3: Mechanisms and categorisation of NDM causing genes

Gene	Inheritance	Mechanism	Category
EIF2AK3	Autosomal recessive	Dysfunction of a critical ER stress sensor which regulates the unfolded protein response	Beta-cell ER stress
INS	Autosomal dominant	Missense variants affecting key residues prevent the formation of disulphide bridges. Results in accumulation of misfolded insulin protein in the ER, which triggers acute ER stress and beta-cell death through apoptosis	Beta-cell ER stress
ABCC8	Autosomal dominant or recessive	Defect in beta-cell potassium channel subunit. Gain-of-function variants prevent the channel from depolarising to enable insulin secretion.	Functional defect
KCNJ11	Autosomal dominant	Defect in beta-cell potassium channel subunit. Gain-of-function variants prevent the channel from depolarising to enable insulin secretion.	Functional defect
GCK	Autosomal recessive	Defect in glucose sensor, uncoupling insulin secretion from blood glucose level.	Functional defect
INS	Autosomal recessive loss of function	Inactivation of both alleles of the insulin gene leads to complete loss of insulin.	Functional defect
PTF1A	Autosomal recessive	Pancreatic agenesis; failure of pancreatic development *in utero*	Pancreatic developmental defect

PDX1	Autosomal recessive	Pancreatic agenesis; failure of pancreatic development *in utero*	Pancreatic developmental defect
GATA6	Autosomal dominant	Pancreatic agenesis; failure of pancreatic development *in utero*	Pancreatic developmental defect
GLIS3	Autosomal recessive	Mechanism not fully elucidated but loss of this transcription factor likely results in failed beta-cell development *in utero*.	Other
IER31P1	Autosomal recessive	Gene function is unclear, likely that mutations result in increased apoptosis.	Other
SLC19A2	Autosomal recessive	Defect in thiamine transporter protein.	Other
6q24	Paternal uniparental disomy (UPD), duplication of paternal allele, loss of methylation of maternal allele	Methylation defect. Mechanism not yet elucidated but likely results in deregulation of insulin secretion.	Other

ESM Fig. 1:

(a) Frequency distribution plot to show patients ages at sampling (w). Bin size: 104w (2y)

(b) Plot to show age at sampling (w) of patients who were positive for at least one islet autoantibody (n=13, median 13w, IQR 5.5-118) or negative for all three (n=220, median 21w, IQR 8-142.5, p=0.5).

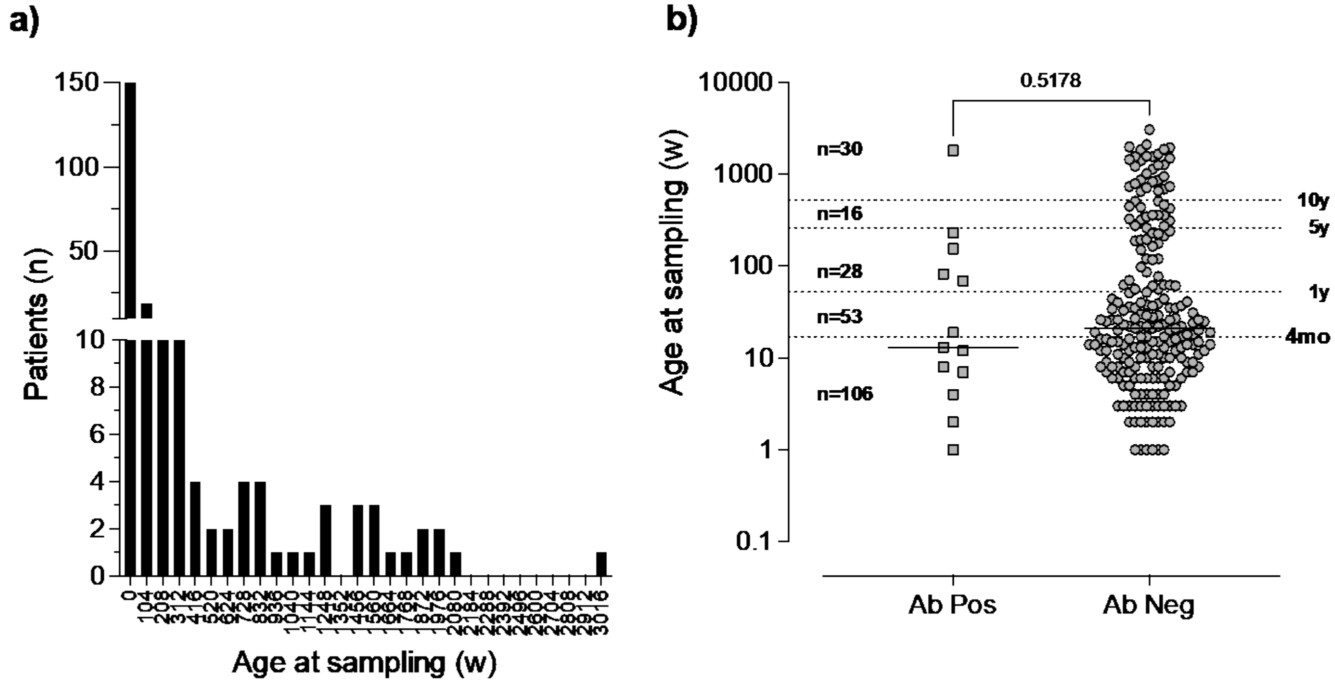

ESM References

[1] Oram RA, Patel K, Hill A, et al. (2016) A Type 1 Diabetes Genetic Risk Score Can Aid Discrimination Between Type 1 and Type 2 Diabetes in Young Adults. Diabetes Care 39(3): 337-344. 10.2337/dc15-1111
